# Professional Mental Health Help-Seeking Amongst Afghan and Iraqi Refugees in Australia: Understanding Predictors Five Years Post Resettlement

**DOI:** 10.3390/ijerph19031896

**Published:** 2022-02-08

**Authors:** Ana-Marija Tomasi, Shameran Slewa-Younan, Renu Narchal, Pilar Rioseco

**Affiliations:** 1 School of Psychology, Western Sydney University, Sydney 2000, Australia; 17690557@student.westernsydney.edu.au (A.-M.T.); R.Narchal@westernsydney.edu.au (R.N.); 2 Mental Health, Translational Health Research Institute, School of Medicine, Western Sydney University, Sydney 2560, Australia; S.Younan@westernsydney.edu.au; 3 Centre for Mental Health, Melbourne School of Population and Global Health, University of Melbourne, Melbourne 3053, Australia; 4 Australian Institute of Family Studies, Melbourne 3006, Australia

**Keywords:** refugees, mental health, help-seeking, physical health, structural barriers, trauma exposure, PTSD, acculturation, discrimination, privacy

## Abstract

The current longitudinal study sought to identify predictors of professional help seeking for mental health problems amongst Afghan and Iraqi refugees five years post-settlement utilising the Building a New Life in Australia dataset (BNLA). Data were collected via face-to-face or phone interviews across five waves from October 2013 to March 2018. Afghan and Iraqi born refugees numbering 1180 and over 18 years of age with a permanent humanitarian visa were included in this study. The results suggest differences in help-seeking behaviors amongst the two ethnic groups. Amongst the Afghan sample, older adults with high psychological distress were more likely to seek help, while living in regional Australia, not requiring interpreters, and knowing how to find out information about government services were related to lower likelihood of help-seeking. Within the Iraqi sample, poor overall health and knowing how to find out about services were related to a greater likelihood of help-seeking, while fewer financial hardships decreased the likelihood of help-seeking. Amongst those with probable PTSD, disability was associated with an increased likelihood of help-seeking while experiencing fewer financial hardships and living in regional Australia resulted in a lower likelihood of help-seeking in this group. These results have implications for promotional material and mental health interventions, suggesting that more integrated services tailored to specific characteristics of ethnic groups are needed.

## 1. Introduction

The world is currently experiencing unprecedented challenges due to the rapid rise in displaced individuals worldwide, with numbers rising from 79.5 million at the end of 2019 [1] to 82.4 million in 2020, highlighting the magnitude of the growing problem [2]. In Australia, refugees from Afghanistan and Iraq have featured in the top five source countries for resettlement since 2016 [3]. Refugees are defined as individuals that are “unable or unwilling to return to their country of origin owing to a well-founded fear of persecution for reasons of race, religion, nationality, membership of a particular social group or political opinion” [4] (p. 3). Between 2018 and 2019 Australia became home to 18,750 refugees under the Humanitarian Visa Program with an additional 12,000 places allocated for individuals displaced by the conflict in Syria and Iraq [3]). Although, these numbers have been impacted by the current global pandemic, they are likely to be reinstated post COVID-19 with additional attention required for those affected by the current political upheaval in Afghanistan [5].

Resulting from exposure to significant trauma in the context of war, difficult migration journeys, and post-migration stressors, refugees often have high rates of psychological distress and mental health disorders [6,7,8]. A systematic literature review by Bogic et al. (2015) found that rates of depression ranged from 2.8% to 80%, rates of post-traumatic stress disorder (PTSD) ranged from 4.4 to 86%, and rates of anxiety ranged from 3% to 88%, with prevalence rates typically over 20% [9]. This is significantly higher than the rates of PTSD, anxiety, and depression reported within the general Australian population (4.4%, 13.1%, and 10.4%, respectively) [10,11].

Considering the high rates of mental health disorders, professional help-seeking, defined as seeking assistance from qualified mental health professionals to manage and treat these disorders, has been low [12,13]. For example, in Europe, a systematic review found that professional help-seeking amongst refugees and asylum seekers with mental health problems ranged from 8.8% to 26% [14]. Similarly, Australian research on Iraqi and Afghan refugees reported only 36–37% of participants received professional help for emotional problems [15]. Another study on Afghan refugees found that only 4.6% of participants with PTSD symptoms disclosed utilising specialist trauma and torture mental health services [16]. By comparison, within the general Australian population, 46% of individuals with mental health problems reported seeking professional help [17].

Anderson’s health care utilisation model can be used to articulate factors that may influence an individual’s utilisation of health care services [18]. The model proposed the following group of factors as influential: predisposing factors (socio-demographic characteristics), enabling factors (income, knowledge of health systems, travel, and waiting time), need (individuals health status), and external factors (community characteristics and interactions with health care providers) [19].

In line with Anderson’s model, previous research has also identified demographic characteristics as influential in determining professional help-seeking [18,20,21]. Although inconsistent amongst refugee groups, age has been positively associated with professional help-seeking within the general population [22,23,24]. Similarly, females across both the general and refugee populations are more likely to discuss mental health, recognise symptoms and seek professional help [25]. Higher education has also been positively correlated with professional help-seeking within the general population, although this association has been inconsistent amongst refugees [15,26]. Proficiency in the host country’s language has been considered a strong predictor of help-seeking across all populations [27,28]. For example, research on Latino and Asian Americans found that limited English-speaking proficiency resulted in delays in treatment, lower perceived need, and reduced utilisation of mental health services [27]. Research has also found variability in help-seeking rates and endorsement of traditional, informal and semi-formal sources of help amongst different ethnic groups [29,30].

Alongside demographic factors, structural barriers may impede utilisation of mental health services [31,32]. Structural barriers can include poor mental health literacy, defined by Jorm et al. (1997) as “the knowledge and beliefs about mental health disorders which aid their recognition, management or prevention” [33] (p. 182). Refugees often arrive to the host country with poor mental health literacy and limited understanding of the new health care system, available services, the role of mental health professionals, and pathways to obtaining health care [20,21,28]. This is further compounded by financial constraints resulting from not yet securing employment and having a multitude of unmet basic needs, thus reducing the importance placed on help-seeking for mental health problems [20,34,35]. Financial costs can also prevent refugees from seeking specialist services not covered by Medicare and restrain travel due to transportation expenses [21,34,36]. Other structural barriers may include difficulties with obtaining appointments, finding childcare, and taking time off work for those employed [21]. For refugees, extended waiting periods for appointments could be further exacerbated if interpreters are required and are difficult to obtain, especially for minority ethnic groups [28]. Once interpreters are secured, additional challenges may arise, including trusting interpreters and mental health professionals to keep information confidential [20]. 

Similarly, experiences of discrimination may also influence help-seeking [37]. Discrimination refers to unfair treatment due to ethnicity, age, gender, disability, sexual orientation, or marital status [38]. For example, a study on Tamil refugees in Canada found that 11% of participants experienced racial discrimination during encounters with health care professionals, resulting in decreased use of these services [39]. 

Acculturation is the process by which migrants adjust their values, beliefs, behaviors, and cultural practices to that of their host country’s culture [40,41]. This process, therefore, involves learning the host nation’s language, navigating new social systems, and familiarising oneself with new norms and ways of living [42]. Refugees’ level of acculturation is likely to influence professional help-seeking. Research on Laotian and Cambodian refugees in America, for example, found that a higher level of acculturation to American mainstream culture was correlated with more positive attitudes towards help-seeking, greater openness when discussing psychological problems, and increased confidence in mental health professionals [25].

It should be noted that despite the increasing focus on help-seeking in refugee populations, findings remain inconsistent, and samples are often small, nonrandom, and are not representative of the population [15]. Additionally, most research has focused on recently arrived refugees, and limited longitudinal studies are available. Consequently, the following study utilises data from the Building a New Life in Australia database (BNLA) [43] to determine the predictors of professional help-seeking for mental health problems within two of the largest refugee groups in Australia: The Afghan and Iraqi population [3]. The BNLA study is the first and most comprehensive longitudinal cohort study in Australia to follow refugees and their families through the first five years of their resettlement journey. The research from the following study is necessary to inform policy makers and service providers on how to best support refugee populations and improve their overall mental health and wellbeing. Additionally, this research can also help clinicians better understand methods to promote engagement with the refugee population.

The aim of the current study was to identify the predictors of professional help-seeking for mental health problems amongst Afghan and Iraqi refugees five years post-resettlement. Informed by Anderson’s model and previous literature, socio-demographic factors, mental health symptomology, general health, trauma exposure, structural barriers, acculturation, and trust and discrimination were included as predictors of interest [18]. Considering the Afghan and Iraqi sub-samples separately, the following was hypothesised:

1. Older age, female gender, higher levels of education, English-speaking proficiency, and residing in a major city would be associated with increased professional help-seeking;
2. Poor overall health and trauma exposure would be associated with greater levels of professional help-seeking;
3. Structural barriers including financial hardship, lack of knowledge about public transport and government services, not receiving an interpreter when needed, and not having an Australian driver’s license would be associated with lower levels of professional help-seeking;
4. Greater acculturation to the mainstream Australian culture would be associated with greater levels of professional help-seeking;
5. Lower trust in government services and experiences of discrimination would be associated with lower levels of professional help-seeking;
6. Finally, amongst the combined Afghan and Iraqi sub-samples who met criteria for PTSD, the following is hypothesised: the association between poorer health, trauma exposure, structural barriers, acculturation, trust, discrimination, and professional help-seeking would be stronger.

## 2. Method

### 2.1. Participants

A total of 1180 adult participants from the BNLA database were included in this study, 773 were from Iraq and 407 were from Afghanistan. The full BNLA sample consisted of humanitarian migrants who arrived in Australia between May and December 2013 (offshore visa) or received their permanent visa during this time (onshore visa). Participants were recruited three to six months after being granted their permanent visa. Using the Australian Settlement Database, “migrating units”—a person or family named on the visa application—were randomly selected from 11 sites across Australia, including major cities and regional areas. These 11 sites were selected based on the number of eligible participants in each site and accounted for almost 92% of humanitarian arrivals in Australia at the time. The BNLA sample is representative of humanitarian migrants who arrived or were granted their permanent visa during this period.

### 2.2. Procedure

An ethics exemption was obtained from the Human Research Ethics Committee at Western Sydney University on the basis that the study would be using pre-existing data. Permission was granted from the Department of Social Services to access the BNLA dataset.

Participants for the BNLA study were recruited based on the principal applicant (PA) on the humanitarian visa. Once PA consented to participate, family members known as secondary applicants (SA) who were over the age of 15 and residing with the PA were also invited to participate. The BNLA dataset was collected annually across five waves from October 2013 to March 2018 via face-to-face (Waves 1, 3 and 5) or telephone interviews (Waves 2 and 4). Questionnaires were translated into 14 languages and consisted of settlement related measures.

### 2.3. Measures

#### 2.3.1. Criterion

**Professional Help-Seeking.** This variable was assessed at Wave 5 using the following question: “In the last 12 months have you received help from a professional, such as a doctor, counsellor or psychologist to help you deal with emotional problems”. Response options included “yes”, “no, I didn’t need it”, and “no, I couldn’t get it” and were coded into “yes” and “no” in line with previous research (earlier waves of the BNLA only included options “yes” and “no”).

#### 2.3.2. Predictors

**Socio-Demographic Characteristics.** Age, gender, country of birth, and highest completed education pre-arrival were all collected at Wave 1 and derived for the following waves. English-speaking proficiency and area of residence were collected at Wave 4. Country of birth acted as a proxy for nationality and only participants from the two largest source countries, Afghanistan and Iraq, were included in this study. Location was categorised into “major cities” or “regional Australia”. Education was categorised into “never attended school”, “9 years or less of school”, “10 or more years of school” and “post-school education”, whilst English-speaking proficiency was categorised into “not at all”, “not well” and “well/very well”.

**General Health.** At Wave 4, participants were asked “*Overall, how would you rate your health during the past 4 weeks?*”. Responses were recorded on a 6-point Likert scale ranging from “excellent” to “very poor” but recoded into “good”, “fair”, and “poor”. The question “*Do you have a disability, injury or health condition that has lasted or is likely to last 12 months or more?*” was also asked in Wave 4 with response options “yes” and “no”.

**Mental Health**. The Kessler Screening Scale for Psychological Distress (K6) and the PTSD-8 Scale were collected at Wave 4 [44,45]. K6 is a six-item measure assessing psychological distress and possible presence of mental illness within the last four weeks. Responses were recorded on a 5-point Likert scale ranging from “none of the time” to “all the time”. Total scores ranged from six to thirty with higher scores suggesting greater psychological distress. The PTSD-8 is an eight-item scale derived from the Harvard Trauma Questionnaire (HTQ) to assess for symptoms of PTSD within the last week. Participants who answered “sometimes” or “most of the time” to at least one item in the three categories of symptoms (intrusions, avoidance, and hypervigilance) were deemed to have probable PTSD. Both scales have been validated cross-culturally [46,47].

**Exposure to Traumatic Events.** Participants at Wave 3 were provided with a list of nine possibly traumatic events and asked to select the ones they experienced prior to arrival in Australia. Examples included the following: combat exposure and torture. Responses were recoded into five categories ranging from “no response” to “4 or more events”.

**Structural Barriers.** Questions available in the BNLA dataset that constituted structural barriers included financial hardship, knowledge of transport and government services, availability of interpreters when needed, and having a current driver’s licence. *(1)* Financial hardship was assessed using the item “*In the last 12 months have any of the following happened to you because you didn’t have enough money*”. Eight response options were provided (e.g., could not pay the rent or mortgage payments on time) and later recoded into five categories ranging from “no financial hardship” to “4+ hardships”; *(2)* ability to use public transport and find out about government services was measured using the question “*We are interested to know how you are getting on with things like transport, accessing information and other day to day activities. If you had to, would you know how to… use public transport, find out what government services and benefits are available”.* Responses were indicated on a 4-point Likert scale ranging from “would know very well” to “wouldn’t know at all”; *(3)* Receiving interpreting assistance was measured using the item “*In the last 12 months, how often were you able to get interpreting assistance when you needed it?*”. Responses were recorded on a 4-point Likert scale ranging from “always” to “never”, and a response option of “haven’t needed interpreting assistance” was also included. *(4)* Finally, “*Do you have a current Australian driver’s licence (including provisional licence)?*” was asked and response options included “yes” and “no”. Data from Wave 4 were used for all variables.

**Acculturation.** Several questions from the survey were utilised as proxies for acculturation. Firstly, length of residency was obtained from the difference between time of arrival and interview date and was derived for the following waves. Responses were recoded into “2 years”, “3 years”, and “4+ years”. Secondly, community support was measured at Wave 4 using the item “*Do you feel that you have been given support/comfort in Australia from (a) Your national or ethnic community (b) Your religious community (c) Other community groups*”. Response options included “yes”, “sometimes”, and “no”. Support from own religious and ethnic community were combined into one variable “support from own community”. Thirdly, friends in Australia were assessed at Wave 4 using the question “*Would you say your friends in Australia are… (a) mostly from my ethnic religious community (b) mostly from other ethnic religious communities (c) a mixture (d) Do not have any friends in Australia yet*”. Responses were recoded into “mostly own ethnic/religious”, “mostly other community/mixture” and “no friends in Australia yet”. Finally, endorsing the item “*getting used to life in Australia*” on the question “*have any of the following been a source of stress in your life in the last 12 months*” indicated possible acculturative stress and was measured at Wave 4. Response options included “not selected” and “selected”.

**Discrimination.** At Wave 4, participants were asked “In the last 12 months, do you think you have been discriminated against, stopped from doing something or been hassled or made to feel inferior, because of your ethnicity, religion or skin color?”. Response options included “yes” and “no”.

**Trust.** Assessed at Wave 4 with the question “Now thinking about government services (e.g., Medicare, Centrelink, public housing), have you experienced any of the following in the last 12 months?… was afraid that my information would not be kept private”. Response options included “no”, “yes”, “haven’t used government services”, and “no response”.

### 2.4. Statistical Analysis and Design

Employing a longitudinal design, statistical analyses were conducted using SPSS version 28.0. The last observation carried forward a method that was utilised when data were unavailable at Wave 4 [48]. Missing data were removed on an analysis-by-analysis basis or a non-response category was included for sensitive questions where missing data were expected. Descriptive analyses included frequencies and proportions for categorical variables and means and standard deviations for continuous variables. Initially, multivariable logistic regressions were conducted separately for the Afghan and Iraqis in blocks of variables based on Anderson’s model: socio-demographics, health, trauma, structural barriers, acculturation, trust, and discrimination [18]. Following this, full models were created including theoretically relevant and statistically significant variables from each block. For parsimony, final models excluded the least significant variables once controlling for all relevant variables from the blocks [49]. Finally, a multivariable logistic regression was conducted with the PTSD sub-sample and only variables in the full model were utilised due to the limited sample size. Given that more than one member of a family could be included in the sample, models were adjusted for clustering at the household level.

## 3. Results

### 3.1. Participant Descriptives

Table 1 presents participants’ descriptive statistics.

Afghan participants were significantly younger (*p* < 0.001), less educated (*p* < 0.001), and more likely to live in regional Australia (*p* < 0.001) compared to those from Iraq. No differences in English speaking proficiency or marital status were noted between groups. Participants from Afghanistan reported better self-rated health (*p* < 0.001), less psychological distress (*p* < 0.001), were less likely to have PTSD (*p* < 0.001) or a disability (*p* < 0.001), and experienced fewer traumatic events (*p* < 0.001) compared to participants from Iraq. In terms of structural barriers Afghan respondents had less knowledge about government services (*p* < 0.001) and were less likely to obtain an interpreter when needed (*p* < 0.001). However, fewer Iraqi refugees held a driver’s licence (*p* = 0.003), and they faced more financial hardships (*p* < 0.001) than their Afghan counterparts. Moreover, despite Afghan refugees having resided in Australia for longer (*p* < 0.001), they were less likely to receive support outside of their own community compared to those from Iraq (*p* = 0.030). No other differences were noted between groups regarding support or friends. Refugees from Afghanistan reported more privacy concerns and were less likely to utilise government services in comparison to Iraqi refugees (*p* < 0.001). Differences in experiences of discrimination were non-significant between groups (*p* = 0.274).

Finally, 34.4% of refugees from Afghanistan (95% CI 31.74, 37.31) and 33.8% of refugees from Iraq (95% CI 30.55, 37.33) received professional help for emotional problems. Amongst those that met criteria for PTSD, 47.3% received professional help (95% CI 41.32, 53.49).

### 3.2. Multivariable Analysis

#### 3.2.1. Afghan Sample

Within the block analysis, socio-demographic characteristics such as being female and older were positively associated with professional help-seeking, while those living in regional Australia were less likely to receive help. Refugees reporting poorer physical and mental health were more likely to have received professional help. In contrast, refugees facing fewer structural barriers were less likely to receive professional help. Specifically, having fewer financial hardships, not requiring interpreting assistance, having a driver’s licence, and knowing how to find out about available government services were all associated with a lower likelihood of seeking help. Measures of acculturation, including not having any friends in Australia and finding the process of getting used to life in Australia stressful, were both associated with increased likelihood of receiving professional help. In the full model, only older age and higher psychological distress were positively associated with help-seeking. Refugees who lived in regional Australia did not require interpreting assistance and who were able to find out about available government services were less likely to receive help (Table 2).

#### 3.2.2. Iraqi Sample

Within the Iraqi block analysis, older refugees were more likely to receive professional help while those with good English-speaking proficiency were less likely to receive help. Having a long-term disability or illness was also associated with increased likelihood of receiving professional help. Structural barriers such as having fewer financial hardships resulted in reduced professional help-seeking, while refugees that were able to receive an interpreter were more likely to seek professional help. Within the full model, poorer physical and mental health, alongside being able to find available government services, increased the likelihood of receiving help. In contrast, participants with fewer financial hardships were less likely to receive help (Table 2).

### 3.3. PTSD Sample

Amongst those with probable PTSD, having a disability or long-term illness was significantly associated with increased professional help-seeking, whereas having fewer financial hardships and living in regional Australia resulted in reduced help-seeking (Table 3).

## 4. Discussion

This study sought to understand the factors associated with professional help-seeking for mental health problems within Iraqi and Afghan refugee populations in Australia, five years post-settlement. Factors of interest included socio-demographic characteristics, health, trauma exposure, structural barriers, acculturation, trust in government services, and experiences of discrimination, in line with Anderson’s health care utilisation model [18]. The findings partially support the hypotheses, with differences noted between the two ethnic groups. Understanding these factors is crucial to developing suitable services tailored to the needs of refugees and enhancing their ability and willingness to access and engage with these services. Additionally, considering the increased rates of trauma-related mental illness amongst refugee populations, understanding the factors that promote professional help-seeking is critical in reducing the burden of mental health disorders on Australian communities [12,26].

Significant socio-demographic factors in the full model included age and living in regional Australia, albeit only in the Afghan sample. No associations were found between professional help-seeking and education level or being married. In the block analysis within the Afghan group, consistent with prior studies, older age and female gender were positively associated with professional help-seeking, while living in regional Australia held a negative association with help-seeking [15,25,50]. Similarly, amongst Iraqi refugees, being older was positively related to help-seeking; however, having good English-speaking proficiency resulted in a lower likelihood of seeking help. This finding contradicts previous research, which has suggested that refugees with poor English-speaking proficiency were less likely to receive professional help and have greater difficulties accessing services [28,51]. These results suggest that extra efforts are needed to promote mental health services amongst younger refugees and males, and additional mental health services targeting refugees in remote areas of Australia are needed. Further research in understanding why those with good English-speaking proficiency were not receiving professional help would be beneficial.

Amongst health and trauma related factors as hypothesised, within the full model, poorer self-rated health, greater psychological distress, and presence of a disability all increased the probability of seeking professional help while meeting criteria for PTSD held no association with help-seeking. Trauma exposure was significant at the block analysis level but only for the Iraqi sub-sample. Again, differences were noted between the two ethnic groups. Specifically, only psychological distress was consistently associated with professional help-seeking across both the Afghan and Iraqi refugee groups. Disability or presence of a long-term illness held no association with professional help-seeking amongst the Afghan sub-sample but was positively associated in the Iraqi group. These findings are aligned with Anderson’s model (1995), which identified need as an important predictor of health care utilisation [18]. Interestingly, previous research on Afghan refugees found that higher rates of disability and better self-recognition of mental health symptoms were correlated with increased help-seeking from mental health professionals [16]. The association between physical health and professional help-seeking may be explained by the higher rates of somatic symptoms reported within refugee groups. These individuals may be seeking help for physical complaints and consequently receiving help for mental health problems [52]. The contrasting findings regarding disability in the Afghan sample could possibly be influenced by better overall health and lower rates of disability reported by Afghan participants compared to Iraqi participants. Finally, differences between ethnic groups in their help-seeking behaviours are consistent with previous research and highlight the importance of targeted mental health promotions that are culturally attuned to the characteristics of specific refugee groups [16]. Other clinical implications of these findings are that general practitioners may need specifically targeted training in recognising symptoms of poor mental health within this population and referral options to mental health specialists tailored to the needs of refugee groups.

The hypotheses related to structural barriers were also partly supported, and again, differences noted between the two groups. Specifically, amongst the Iraqi sub-sample in the full model, having fewer financial hardships was related to a decreased likelihood of receiving professional help, while, as hypothesised, knowing how to find out about available government services was associated with a greater likelihood of receiving professional help. In contrast, amongst the Afghan sub-sample, knowing fairly well how to find out about government services and not needing interpreting assistance was related to a lower likelihood of seeking professional help. Findings from the block analysis also suggested Afghan refugees with an Australian driver’s license were less likely to receive professional help. No associations were found between knowing how to use public transport and professional help-seeking. These findings are contrary to previous research, which found financial hardship to be a significant barrier to help-seeking for most refugees as they were unable to pay for treatment or travel costs and often prioritised financial security over receiving help for mental health disorders [20]. A systematic review by Byrow et al. (2020) also found limited access to interpreters and understanding how to access available services was related to reduced help-seeking and longer durations of mental health disorders being left untreated [20]. A possible explanation for our contrasting results could be that refugees who were experiencing financial difficulties, language barriers, and difficulties navigating the new social systems were receiving support from other social services. It is possible that they were identified as needing psychological help in the context of other support services and had been linked with mental health professionals [53]. These findings imply that additional efforts are required to promote mental health services amongst refugees who are not in contact with other social services. They also again highlight the importance of targeted mental health promotion and services that focus on refugee groups separately based on cultural background.

When acculturative factors were taken into account in the full model, no factors were significantly associated with professional help-seeking. However, within the block model, contrary to predictions, those who found the acculturation process stressful and had no friends were more likely to seek professional help, albeit only in the Afghan sub-sample. No significant associations were found between time spent in Australia, receiving support from either one’s own ethnic community or other communities and professional help-seeking. These findings are inconsistent with previous research, which has found that refugees who were more acculturated to the host culture were more likely to endorse seeking professional support for mental health problems [29]. However, previous studies have found that less acculturated refugees who experienced greater acculturative stress where more likely to have poorer mental health [53,54]. Therefore, it may be plausible in our study that those refugees who were less acculturated were experiencing poorer mental health and, thus, as suggested in Anderson’s health care utilisation model, had a greater need for professional help [18]. These findings insinuate the need for additional services for refugees during the settlement period even five years post arrival to assist with adjusting to the host country and fostering social support. It is also important to mention that, given the study’s design, time spent in Australia amongst our sample was highly homogenous. Future research incorporating a longer time span with greater sample variability is required to further examine the role of time in the host country as a predictor of help-seeking.

Both trusting that government services would keep information private and experiencing discrimination, were not significantly associated with receiving professional help. However, previous research on adolescent refugees in Australia found that lower trust in health professionals was related to fears that information would be disclosed to other community members, resulting in lower rates of professional help-seeking. Similarly, research has suggested that experiencing discrimination in the host country was associated with both poorer mental health and a reluctance to seek professional help [55,56]. Within our study, a very small number of participants reported experiencing discrimination; thus, this association may require further exploration with a larger sample size. Future studies could include open ended questions in order to develop a deeper understanding of refugees’ experiences of discrimination and its influence on help-seeking.

Finally, amongst the sub-sample who met criteria for PTSD, only having a long term-disability or illness was positively related to receiving professional help. However, refugees with PTSD who had fewer financial hardships and lived in remote parts of Australia were less likely to receive professional help. These findings again highlight the possibility that refugees are being identified as needing help when they access other social services and health professionals and are then referred onto mental health professionals. The implications of these results are that more integrated and culturally informed services are required for this population. Additionally, it may be beneficial to promote mental health services amongst refugees who are performing well in other social and health domains apart from mental health. Help-seeking was lower among refugees with likely PTSD in regional areas, highlighting the need to improve availability and access to mental health services outside of major cities.

This study has a number of limitations. Firstly, as self-report measures were utilised, results may have been impacted by social desirability and recall bias [57]. Responses may have also been affected by issues around translation of surveys and transcultural bias [58]. A limitation of our quantitative design is that it is not clear whether participants fully understand subjective concepts, such as discrimination. Additionally, variables such as community support may have been influenced by perceptions of need for support rather than acculturation. Despite some limitations, the large sample size representative of the leading groups of refugees in Australia is a major strength of this study, as these results can be utilised to improve the resettlement outcomes of a large proportion of refugees in Australia. Future research may wish to extend these findings by utilising a more extensive measure of professional help-seeking. Future research including open-ended questions, or a mixed-method design would be beneficial. This would provide the opportunity to ask follow-up questions and, therefore, gain a better in-depth understanding of the reasons for selecting certain options. Additionally, incorporating qualitative methodologies would contribute to our understanding of the underlaying cultural characteristics that my influence help-seeking behaviors amongst these two populations.

## 5. Conclusions

This study was amongst the first to explore professional help-seeking for mental health problems five years post-settlement amongst two of the largest resettled groups of refugees in Australia. The findings showed significant differences between the two ethnic groups and suggested socio-demographic variables, poorer overall health and structural barriers all predicted professional help-seeking, albeit differently within the two refugee groups. These findings have significant implications for policy makers and service providers who aim to support this group during the resettlement period.

## Figures and Tables

**Table 1:** Descriptive statistics of predictor variables by country of birth, PTSD sample and total sample.

Variables		Afghanistan % (*N* = 407) (*n*; *SE)*	Iraq % (*N* = 773) (*n*; *SE*)	PTSD % (*N* = 281) (*n*; *SE*)	Total % (*N* = 1180) (*n*; *SE*)
Socio-demographics					
	Age	38.72 (379; 0.600)	43.51 (743; 0.536)	45.14 (281; 0.679)	41.89 (1122; 0.414)
Gender	Male	56.3 (229)	52.5(406)	45.9 (129)	53.8 (635)
	Female	43.7 (178)	47.5(367)	54.1 (152)	46.2 (545)
Married/Partnered	No	31.7 (120)	31.0 (229)	31.3 (88)	31.3 (349)
	Yes	68.3(258)	69.0 (509)	68.7 (193)	68.7 (767)
Education Level	Never attended School	40.6 (164)	6.0 (55)	11.5 (32)	17.9 (210)
	9 years or less	45.5(184)	39.5 (304)	44.6 (124)	41.6 (488)
	10 years or less	11.4(46)	32.0 (246)	22.3 (62)	24.9 (292)
	Post-school education	2.5 (10)	22.5 (173)	21.6 (60)	15.6 (183)
Location	Major cities	86.1 (327)	99.6 (740)	94.7 (266)	95.0 (1067)
	Regional Australia	13.9 (53)	0.4(3)	5.3 (15)	5.0 (56)
English Proficiency	Not at all	18.9 (71)	14.3 (105)	21.0 (59)	15.8 (176)
	Not well	44.4 (167)	45.4 (334)	49.8 (140)	45.1 (501)
	Well/Very well	36.7 (138)	40.2 (296)	29.2 (82)	39.1 (434)
Health					
Self-rated Health	Good	70.6 (266)	51.2 (377)	29.2 (82)	57.8 (643)
	Fair	15.1 (57)	26.0 (191)	32.4 (91)	22.3 (248)
	Poor	14.3 (54)	22.8 (168)	38.4 (108)	19.9 (222)
Kessler6 Total Score		11.37 (377; 0.294)	13.07 (735; 0.237)	18.43 (0.374)	12.49 (1112; 0.187)
PTSD	Unlikely to have PTSD	85.4 (321)	69.2 (507)	-	74.7 (828)
	May have PTSD	14.6 (55)	30.8 (226)	100 (281)	25.3 (281)
Long Term Illness/Disability	Yes	28.5 (106)	43.0 (305)	61.5 (169)	38.0 (411)
	No	71.5 (266)	57.0 (405)	38.5 (106)	62.0 (671)
Trauma Exposure					
Traumatic Events	No response	22.7 (82)	8.0 (56)	7.2 (19)	13.0 (138)
	1	35.7 (129)	21.6(152)	24.2 (64)	26.4 (281)
	2	15.0 (54)	15.2 (107)	10.2 (27)	15.1 (161)
	3	8.0 (29)	18.6 (131)	15.5 (41)	15.0 (160)
	4 or more	18.6 (67)	36.6 (257)	42.8 (113)	30.5 (324)
Structural Barriers					
Financial Hardship	0	62.3 (236)	52.7 (389)	35.7 (100)	56.0 (625)
	1	12.4 (47)	22.6 (167)	28.2 (79)	19.2 (214)
	2	10.0 (38)	11.9 (88)	14.3 (40)	11.3 (126)
	3	6.9 (26)	7.7 (57)	13.2 (37)	7.4 (83)
	4 or more	8.4 (32)	5.0 (37)	8.6 (24)	6.2 (69)
Got interpreter when needed	Never	18.6 (70)	13.9(102)	3.6 (10)	15.5 (172)
	Some of the time	35.4 (133)	26.0 (191)	22.4 (63)	29.1 (324)
	Most of the time	10.4 (39)	18. 5(136)	20.6 (58)	15.7 (175)
	Always	19.1 (72)	31.4 (231)	41.6 (117)	27.2 (303)
	Haven’t needed	16.5 (62)	10.3 (76)	11.7 (33)	12.4 (138)
Use public transport	Would know very well	61.2 (226)	53.0 (376)	42.5 (117)	55.8 (602)
	Would know fairly well	19.0 (70)	21.9 (155)	26.2 (72)	20.9 (225)
	Would know a little	10.8 (40)	13.4 (95)	16.7 (46)	12.5 (135)
	Wouldn’t know at all	8.9 (33)	11.7 (83)	14.5 (40)	10.8 (116)
Find gov. services	Would know very well	28.8 (106)	34.5 (244)	14.6 (40)	32.6 (350)
	Would know fairly well	19.8 (73)	21.5 (152)	23.4 (64)	20.9 (225)
	Would know a little	20.7 (76)	26.2 (185)	33.6 (92)	24.3 (261)
	Wouldn’t know at all	30.7 (113)	17.8 (126)	28.5 (78)	22.2 (239)
Aus. Driver’s Licence	Yes	84.1 (328)	76.7 (569)	73.5 (202)	79.2 (897)
	No	15.9 (62)	23.3 (173)	26.5 (73)	20.8 (235)
Acculturation					
Time between arrival and interview	2 years	3.7 (14)	3.8 (28)	2.1 (6)	3.7 (42)
	3 years	76.3 (289)	93.5 (695)	92.5 (260)	87.7 (984)
	4 or more	20.1 (76)	2.7 (20)	5.3 (15)	8.6 (96)
Community Support (own)	Yes	26.1 (94)	27.9 (205)	29.4 (82)	27.3 (299)
	Sometimes	29.4 (106)	21.4 (157)	23.3 (65)	24.0 (263)
	No	44.4 (160)	50.7 (372)	47.3 (132)	48.6 (532)
Community Support (other)	Yes	12.3 (43)	18.6 (135)	14.3 (39)	16.6 (178)
	Sometimes	14.8 (52)	13.7 (99)	15.8 (43)	14.0 (151)
	No	72.9 (256)	67.7 (490)	69.9 (190)	69.4 (746)
Friends in Australia	Mostly own ethnic/rel	43.9 (147)	39.2 (260)	43.3 (113)	40.7 (407)
	Mostly mixture/other comm	47.8 (160)	50.8 (337)	41.4 (108)	49.7 (497)
	No friends Aus yet	8.4 (28)	10.1 (67)	15.3 (40)	9.5 (95)
Source of stress-getting used to life in Australia	No selected	92.9 (312)	89.8 (624)	83.0 (224)	90.8 (938)
	Selected	7.1 (24)	10.2 (71)	17.0 (46)	9.2 (95)
Trust					
Privacy	Non response	16.7 (57)	9.9 (71)	9.5 (25)	9.8 (100)
	Yes	9.4 (32)	8.0 (57)	7.6 (20)	5.2 (53)
	No	56.3 (192)	74.3 (532)	69.7 (184)	70.6 (720)
	Haven’t used gov. services	17.6 (60)	7.8 (56)	13.3 (35)	14.4 (147)
Discrimination					
Discrimination	No	91.5 (345)	93.3 (684)	86.7 (242)	92.7 (1029)
	Yes	8.5 (32)	6.7 (49)	13.3 (37)	7.3 (81)

*N =* number of observations in the entire sample; *n* = number of observations in each variable; % is percentage of participants; *SE* = standard error.

**Table 2:** Multivariate analysis by country of birth, including the predictors in blocks (demographics, health, trauma exposure, structural barriers, acculturation, trust and discrimination and the criterion professional help-seeking) and the full model.

		Afghanistan				Iraq			
		Block Model		Full Model		Block Model		Full Model	
		OR	95% CI	OR	95% CI	OR	95% CI	OR	95% CI
Socio-demographics									
	Age	1.04 ***	[1.02,1.07]	1.04 *	[1.00,1.07]	1.02 **	[1.01,1.04]	1.01	[0.99,1.03]
Gender	Male (ref.)								
	Female	1.91 *	[1.17,3.12]	1.20	[0.65,2.23]	1.03	[0.77,1.38]	1.17	[0.83,1.65]
Married/Partnered	No (ref.)								
	Yes	0.79	[0.45,1.40]	-	-	1.11	[0.77,1.62]	-	-
Education level	Never attended School (ref.)								
	9 years or less	1.26	[0.76,2.09]	-	-	1.66	[0.72,3.82]	-	-
	10 years or less	1.16	[0.51,2.62]	-	-	1.74	[0.73,4.14]	-	-
	Post-school education	0.67	[0.10,4.59]	-	-	1.46	[0.59,3.61]	-	-
Location	Major cities (ref.)								
	Regional Australia	0.34 *	[0.14,0.79]	0.19 ***	[0.08,0.48]	1.00	[1.00,1.00]	-	-
English proficiency	Not at all (ref.)								
	Not well	0.79	[0.40,1.59]	1.47	[0.57,3.78]	0.63	[0.38,1.05]	0.62	[0.36,1.07]
	Well/Very well	0.71	[0.30,1.69]	2.56	[0.80,8.23]	0.46 *	[0.25,0.84]	0.55	[0.27,1.14]
*N*		373				728			
Health									
Self-rated health	Good (ref.)								
	Fair	2.09 *	[1.13,3.88]	1.89	[0.89,4.04]	1.54	[0.99,2.40]	1.49	[0.92,2.41]
	Poor	2.57 *	[1.22,5.40]	1.40	[0.54,3.64]	2.12 **	[1.24,3.62]	2.09 *	[1.15,3.79]
Kessler 6 Total Score		1.06 *	[1.01,1.12]	1.11 **	[1.04,1.19]	1.05 **	[1.02,1.09]	1.05 *	[1.01,1.09]
PTSD	No PTSD (ref.)								
	Probable PTSD	0.84	[0.38,1.87]	1.05	[0.41,2.68]	0.98	[0.63,1.51]	1.06	[0.66,1.70]
Long term illness or disability	No (ref.)								
	Yes	1.48	[0.80,2.73]	1.61	[0.80,3.23]	2.51 ***	[1.72,3.64]	2.31 ***	[1.51,3.55]
*N*		371				708			
Trauma Exposure									
	1 (ref.)								
	2	1.04	[0.53,2.04]	-	-	1.85 *	[1.08,3.16]	-	-
	3	0.86	[0.36,2.08]	-	-	2.05 **	[1.23,3.43]	-	-
	4+	1.04	[0.55,1.93]	-	-	2.19 ***	[1.42,3.39]	-	-
	No response	0.72	[0.39,1.33]	-	-	1.53	[0.81,2.88]	-	-
*N*		361				703			
Structural Barriers									
Financial Hardship	4 or more (ref.)								
	0	0.60	[0.25,1.42]	1.37	[0.48,3.91]	0.27 **	[0.12,0.61]	0.41 *	[0.19,0.89]
	1	0.91	[0.32,2.54]	1.18	[0.39,3.57]	0.32 **	[0.14,0.73]	0.39 *	[0.18,0.85]
	2	1.23	[0.42,3.60]	1.98	[0.61,6.40]	0.21 **	[0.08,0.54]	0.21 ***	[0.08,0.52]
	3	0.31 *	[0.10,0.98]	0.49	[0.13,1.87]	0.83	[0.29,2.37]	0.90	[0.31,2.64]
Got interpreter when needed	Never (ref.)								
	Some of the time	1.53	[0.77,3.04]	1.28	[0.55,2.98]	2.20 *	[1.17,4.12]	1.24	[0.64,2.43]
	Most of the time	1.05	[0.38,2.88]	0.91	[0.28,3.01]	1.95	[0.95,3.97]	0.95	[0.41,2.18]
	Always	2.03	[0.93,4.43]	1.27	[0.45,3.58]	3.78 ***	[1.92,7.43]	1.44	[0.65,3.18]
	Haven’t needed	0.31 *	[0.10,0.92]	0.28 *	[0.09,0.86]	1.47	[0.67,3.21]	1.29	[0.56,2.98]
Use public transport	Wouldn’t know at all (ref.)								
	Would know very well	1.10	[0.44,2.78]	-	-	0.69	[0.35,1.35]	-	-
	Would know fairly well	1.38	[0.49,3.90]	-	-	0.67	[0.33,1.35]	-	-
	Would know a little	2.21	[0.80,6.13]	-	-	0.93	[0.46,1.86]	-	-
Find gov. services	Wouldn’t know at all (ref.)								
	Would know very well	0.71	[0.35,1.47]	0.61	[0.27,1.38]	1.50	[0.82,2.74]	2.07 *	[1.15,3.75]
	Would know fairly well	0.46	[0.21,1.02]	0.33 **	[0.15,0.76]	1.48	[0.80,2.75]	1.81	[0.99,3.31]
	Would know a little	0.49 *	[0.25,0.96]	0.52	[0.24,1.15]	1.16	[0.67,2.01]	1.35	[0.78,2.35]
Aus. Driver’s Licence	No (ref.)								
	Yes	0.53 *	[0.28,1.00]	-	-	0.78	[0.50,1.22]	-	-
*N*		362				691			
Acculturation									
Time btw. Arrival and interview	4 or more (ref.)								
	3 years	0.67	[0.36,1.26]	-	-	1.16	[0.33,4.09]	-	-
	2 years	0.37	[0.06,2.16]	-	-	1.76	[0.37,8.44]	-	-
Community Support (own)	No (ref.)								
	Sometimes	1.03	[0.55,1.91]	-	-	1.07	[0.64,1.79]	-	-
	Yes	0.84	[0.41,1.73]	-	-	0.82	[0.50,1.36]	-	-
Community Support (other)	No (ref.)								
	Sometimes	0.82	[0.37,1.83]	-	-	0.67	[0.37,1.22]	-	-
	Yes	0.44	[0.16,1.24]	-	-	1.04	[0.57,1.92]	-	-
Friends in Australia	Mostly mixture/ other comm. (ref.)								
	Mostly own ethnic/rel	1.52	[0.89,2.61]	-	-	1.39	[0.98,1.97]	-	-
	No friends Aus yet	3.09 **	[1.38,6.92]	-	-	1.43	[0.82,2.49]	-	-
Source of stress- getting used to life in Australia	Not selected (ref.)								
	Selected	3.14 *	[1.21,8.18]	2.31	[0.74,7.25]	1.44	[0.86,2.43]	0.87	[0.48,1.58]
*N*		277				646			
Trust									
Privacy	No (ref.)								
	Yes	0.94	[0.31,2.86]	-	-	1.78	[0.92,3.47]	-	-
	Nonresponse	1.20	[0.59,2.46]	-	-	1.46	[0.83,2.56]	-	-
	Haven’t used gov. services	1.29	[0.73,2.29]	-	-	1.15	[0.70,1.88]	-	-
*N*	342					678			
Discrimination									
Discrimination	No (ref.)								
	Yes	0.67	[0.30,1.49]	-	-	1.14	[0.64,2.02]	-	-
*N*		377		331		733		686	

OR = odds ratio; 95% CI = 95% confidence interval; *N* = number of observations. Ref. = reference category. *p* < 0.05 *, *p* < 0.01 **, *p* < 0.001 ***.

**Table 3:** Full model displaying predictors of professional help-seeking within the PTSD sample.

Variables		OR	95% CI
Country of Birth	Iraq (ref.)		
	Afghanistan	1.89	[0.68,5.26]
Socio-demographic			
	Age	0.99	[0.96,1.02]
Gender	Male (ref.)		
	Female	1.35	[0.75,2.42]
English Proficiency	Not at all (ref.)		
	Not well	0.99	[0.44,2.25]
	Well/very well	0.75	[0.21,2.63]
Location	Major cities (ref.)		
	Regional Australia	0.15 *	[0.03,0.82]
Health			
Self-rated Health	Good (ref.)		
	Fair	1.78	[0.61,5.42]
	Poor	1.82	[0.61,5.42]
Kessler 6	Total score	1.05	[0.99,1.11]
Long Term Illness or Disability	No (ref.)		
	Yes	2.74 **	[1.35,5.59]
Structural Barriers			
Financial Hardship	4 or more (ref.)		
Financial Hardships	0	0.27 *	[0.08,0.92]
	1	0.34	[0.10,1.17]
	2	0.32	[0.09,1.24]
	3	0.69	[0.18,2.67]
Got interpreter when needed	Never (ref.)		
	Some of the time	3.35	[0.63,17.68]
	Most of the time	2.49	[0.39,15.67]
	Always	3.42	[0.57,20.70]
	Haven’t needed	3.08	[0.57,16.83]
Find gov. services	Wouldn’t know at all (ref.)		
	Would know very well	1.67	[0.57,4.88]
	Would know fairly well	0.87	[0.37,2.03]
	Would know a little	0.66	[0.31,1.44]
Source of stress- getting used to life in Australia	Not selected (ref.)		
	Selected	1.24	[0.57,2.68]
*N*	260		

OR = odds ratio; 95% CI = 95% confidence interval; *N* = number of observations. Ref. = reference category. *p* < 0.05 *, *p* < 0.01 **.

## Data Availability

Data used in this study are publicly available to approved researchers. Access is managed by the National Centre for Longitudinal Data on National Centre for Longitudinal Data Dataverse.
